# Environmental Kuznets curve for CO2 emissions in Baltic countries: an empirical investigation

**DOI:** 10.1007/s11356-022-19103-3

**Published:** 2022-02-18

**Authors:** Ashim Kumar Kar

**Affiliations:** Faculty of Social Sciences, University of Helsinki, Helsinki, Finland

**Keywords:** CO_2_ emissions, Environmental Kuznets curve, Panel data, Cross-sectional dependency, Heterogeneity, Baltic countries, Q01, Q43, Q53, Q54, Q56, Q57

## Abstract

Recognizing the factors responsible for the gradual increase in greenhouse gas [e.g. carbon dioxide (CO_2_)] emissions is crucial to reduce the detrimental consequences on environmental sustainability and human life. Accordingly, spotting the sectors which contribute the most to CO_2_ emissions and dampen economic growth have become one of the major concerns for policymakers around the globe. Against this background, this paper examines the nexus between economic growth and CO_2_ emissions in three Baltic countries namely Estonia, Latvia and Lithuania. Thus, the study basically checks the validity of the environmental Kuznets curve (EKC) hypothesis by taking into account the role of energy consumption and financial development over the period of 1990–2018. This type of study is highly important for the region in order to comply with the commitments of the Paris Agreement and Sustainable Development Goals of the United Nations. The study first employs appropriate testing procedures and second-generation panel data methods to account for cross-sectional dependency and slope heterogeneity among countries. Applying unit roots and cointegration tests, the study then employed different mean group estimation models and heterogeneous panel causality methods suitable for cross-sectionally dependent and heterogeneous panels. The results of the econometric analyses reveal that the inverted U-shaped EKC hypothesis does not hold in the Baltic countries. But the pollution haven hypothesis is evidenced to hold for these nations. By boosting the CO_2_ emissions figures, again, the study also revealed that higher levels of energy consumption exhibit adverse environmental consequences. Financial development is found to be effective in explaining the variations in the CO_2_ emission figures of the selected countries as well. Causality test results confirm bi-directional causality between economic growth and CO_2_ emissions, energy use and CO_2_ emissions, CO_2_ emissions and financial development, energy use and economic growth as well as between energy use and financial development. Furthermore, country-specific impacts are found to be similar to the corresponding panel estimates. Consistent with the findings, the study finally puts forward some policy-level suggestions. Accordingly, it is recommended that the Baltic countries need to move away from fossil-fuel dependent energy consumption growth policies to mitigate environmental degradation.

## Introduction

Global warming is the most critical environmental challenge of our age. Thus, climate change—an impact of global warming—has become a global threat to sustainable development (Destek and Sarkodie 2019). We know that emission of greenhouse gases (GHGs)—like carbon dioxide, nitrous oxide, methane, sulphur hexafluoride, fluorinated gases—are the key drivers to global warming. Evidently, human activities—including the use of fossil fuel as a source of energy—are also greatly responsible for the gradual increase in GHG emissions, hence for the resultant pollution and global warming (Ali et al. 2017; Rehman et al. 2021). We, however, need to use energy for economic activities. Thus, pollution is in a sense the ultimate price for economic development. Conservation of energy helps mitigate carbon dioxide (CO_2_) emissions, but it might come at the cost of people’s living standard and economic growth (Ali et al. 2017). Hence, it is very important to understand the effects of economic growth on the environment as reducing CO_2_ emissions to control global warming is not a simple issue (Allard et al. 2018; Adedoyin et al. 2020).

The United Nations (UN) has declared 17 Sustainable Development Goals (SDGs) in 2015, which demonstrate the scale and ambition of the 2030 agenda for sustainable development. The 7th SDG, in particular, emphasised access to affordable, reliable and modern energy so that the energy efficiency is doubled and income inequality is mitigated by 2030 (Murshed 2020; Murshed 2021; Acheampong et al. 2021)^1^. Again, the United Nations Framework Convention on Climate Change (UNFCCC) embraced the Paris Agreement (PA) to deal with the danger of climate change (United Nations 2015). Following their ratification of the PA, all three Baltic countries (namely, Estonia, Latvia and Lithuania) have also agreed to fight the global climate change threat which is mostly caused by GHGs like CO_2_, sulphur dioxide (SO_2_), nitrous oxide (NO_2_) and methane (CH_4_). Thus, to fight global warming, these economies are obliged to pursue efforts to reduce the greenhouse gas emissions and to keep the global temperature levels well below 2 °C (preferably up to 1.5 °C) above the pre-industrial level by the end of this century though they have their own national environmental protection and sustainability objectives.

In the literature, the environmental Kuznets curve (EKC) framework is widely used to assess the effects of economic growth on environmental quality. The EKC hypothesis stems from the work of Kuznets (1955) who revealed a non-linear (inversed-U shaped) relationship between per capita income and income inequality^2^. Of late, Grossman and Krueger (1991, 1995), Panayotou (1993), Shafik and Bandyopadhyay (1992) as well as Holtz-Eakin and Selden (1995) reinterpreted the Kuznets’ hypothesis in the context of growing environmental issues. They basically suggested that the pollutants (such as CO_2_, NO_X_ and SO_2_) and output have an inverted U-shaped relation^3^. So, at the early stages of economic growth, environmental degradation rises with output, but declines after reaching a certain threshold (Apergis 2016). In other words, most environmental degradation factors tend to worsen due to economic growth until the average income is at a certain level (Stern 2004). This also suggests that economic growth may improve environmental conditions if appropriate and corrective measures are in place.

Nevertheless, energy is a potential catalyst for socio-economic development (Ali et al. (2017)). But the connection between economy and environment is extremely complex and often controversial. Accordingly, many have criticised the theoretical foundations of the EKC hypothesis that makes empirical studies on this topic debatable. The number of empirical research on this topic, however, is substantial, and many of them have aimed at validating the EKC hypothesis using the income–pollution linkage (see, for example, Al-Mulali et al. 2015; Sarkodie 2018; Sarkodie and Ozturk 2020; Shakib et al. 2021; Wang et al. 2022a; Wang et al. 2022b) 4. Again, while a number of studies have attempted to test the EKC hypothesis via time series analysis, many have also opted for cross-sectional or panel data analysis. Identical cross-section data and similar econometric models have been employed in many empirical papers too. However, reviews of relevant literature shows that there is no consensus on this relationship so far and the results in general are mixed. Some studies conclude that the intensity of pollutant emissions initially increases with the income per capita, but eventually falls after certain threshold point, at least in the case of the developed countries, while other studies claim that such relationship may not be robust for a number of emission pollutants.

Arguably, such mixed results can be due to data aggregation bias—difference between the group-level effects and individual effects—raising questions on the external and internal validity of the results. To be more specific, for example, once data of a number of countries are employed in panel empirical models, one country’s significant income effect could be over-offset by other country’s insignificant income effect and vice versa. As a result, EKC for a pollutant may be non-existent. Again, earlier studies have focused either on individual countries (e.g. China, India, Pakistan. Malaysia, Turkey in Asia; Saudi Arabia, Lebanon in the MENA region; South Africa, Kenya in Africa; Germany, the UK in Europe)—or on different country clusters or regional groups (e.g. OECD, MENA, GCC, SAARC, CEE, Latin America). But many country clusters have not yet been comprehensively covered (e.g. the Baltic region). Again, the literature on the EKC hypothesis has primarily concentrated on SO_2_ and NOx emissions with a few exceptions that concentrated on CO_2_ emissions. But we can quite justifiably extend the literature dealing with the EKC involving CO_2_ emissions and by using methodological advances in panel data econometrics that allow us to accommodate for cointegration in the presence of non-stationary data. This will help us provide a more accurate picture of the existence, or non-existence, of the EKC. Clearly, therefore, there are scopes for further research and to contribute to the extant literature by testing the EKC hypothesis for different countries or country groups.

This study, therefore, focuses on three Baltic countries (Estonia, Latvia and Lithuania) as a case study for a number of reasons described below. First, studying these countries is important because the economic and financial development levels in these countries are mostly similar and they are close neighbours to one another as well as to the Western Europe (Sadorsky 2011). The countries in this region are generally considered to be highly dependent on fossil fuel-based energy sources and electricity import (Zoss et al. 2016). After attaining membership to the European Union (EU), they have now closer economic and political ties with other European countries, which have started renewable electricity utilisation, though the trends have some differences, in the region. Besides, these countries are located amid Russia and huge-energy-consuming Western European countries (e.g. France, Germany), making their strategic location very important. Therefore, studying the existence of the EKC hypothesis in this region is vital both at the country and regional level. In the context of energy demand in the EU as a whole, the issue is of particular consequence (Sadorsky 2011). However, to the best of our knowledge, only a handful of previous studies have specifically focused in this area. From that point of view, this study is one of the first attempts to concentrate only on three Baltic countries in the EKC literature, though these countries have been studied in other country clusters (e.g. OECD, BEST, EMDE countries). Studying the Baltic countries is also important because they agreed to coordinate their environmental policies by following the Paris climate agreement and for the preparation of Nationally Determined Contributions (NDCs) for national and global mitigation action. The region can therefore serve as a useful case study for other countries and provide valuable lessons on the dynamics and processes required for achieving low carbon energy transitions.

The study contributes to the existing literature in a number of ways. First, the study focuses on the Baltic region which is relatively less studied to check the validity of the EKC hypothesis. Second, unlike previous studies, the study uses an analytical framework that involves second-generation estimation methods. They include cross-sectional dependence (CD) test, panel level heterogeneity tests, unit root tests, cointegration tests, panel level causality test and applying several mean group estimators and random coefficient estimators. Needless to say, studies devoid of applying these techniques may result in biased results. Third, in order to make the empirical findings more policy-oriented, the study presents the results of each country separately. This indicates that country-level determinants are important factors in explaining the environmental degradation scenarios. Fourth, the study employs recent data and expands the period of the time-series data to provide further statistical evidence. Fifth, by exploring the effects of financial development and energy use on CO_2_ emissions in the Baltic countries, the paper provides new information on how to mitigate CO_2_ emissions. Thus, the paper adds to the existing knowledge on the inverted U-shaped relationship between economic growth and environmental degradation. Sixth, the results will provide suggestions for policymakers and practitioners and therefore are expected to contribute to the environmental sustainability literature in general.

The rest of the paper is organized as follows. Section 2 discusses relevant literature. Section 3 provides discussions on the econometric methodologies, data and model specifications. Section 4 reports the results of the empirical and robustness analyses. Finally, Section 5 concludes the paper.

## Literature review

Grossman and Krueger (1991) for the first time emphasised that economic growth can control the problem of environmental degradation. Specifically, environmental degradation increases during the early stages of economic growth, but declines once the economy arrives at a threshold. Thus, attaining economic growth is the quickest and definite way to protect and improve the environment. Accordingly, the environmental Kuznets curve (EKC) hypothesis—an inverted U-shaped relationship between GDP growth and CO_2_ emissions—was developed^5^. Testing the validity of the EKC has been one of the main research strands in energy economics literature for decades now. Researchers are interested in investigating the determinants of CO_2_ emissions and impact of different variables on such emissions. Consequently, a significant number of country-level and cross-country studies have explored the subject, and a huge body of literature is already available now. Panel data studies on this topic have employed different variables on an aggregate level and offered diverse results. Country-specific case studies have found diversified findings too. Therefore, the results obtained so far have been controversial and need more investigation. This was also emphasized by Koondhar et al. (2021) that the research on EKC requires more attention, particularly to contribute to academic development and applied methodology.

### Confirmation and non-confirmation of the EKC hypothesis

The early studies of Selden and Song (1994) and Grossman and Krueger (1995) empirically supported the existence of EKC hypothesis between GDP growth and environmental quality. Bartlett (1994) reported the inverted U-shaped relationship between per-capita income and per-capita CO_2_ emissions indicating the turning point of the EKC. Later, Galeotti et al. (2006) confirmed the inverted U-shaped relationship between these variables having reasonable turning points in the OECD countries, but not for non-OPEC and non-OECD countries. Apart from them, many of the earlier studies have confirmed the existence of an inverted U-shaped relationship between income and environmental degradation (i.e. the EKC hypothesis). For example, a recent study of Alola and Ozturk (2021) confirmed the EKC hypothesis for the USA, but at the detriment of risk to investment, Sarkodie and Ozturk (2020) confirmed it for Kenya, Baloch et al. (2020) validated it for OECD countries, Destek and Sarkodie (2019) confirmed it for 11 newly industrialised countries, Shujah-ur-Rahman et al. (2019) for Pakistan, Aslan et al. (2018a, b) for the USA, Sarkodie and Strezov (2018) for 4 countries (Australia, China, Ghana and USA), Katırcıoglu (2017) for Turkey, Ali et al. (2017) for Malaysia, Ahmed et al. (2016) for 24 European countries, Zhu et al. (2016) for 5 ASEAN countries, Bilgili et al. (2016) for 17 OECD countries and Al-Mulali et al. (2016) for 107 countries (both the short run and the long run). Among others, Al-Mulali et al. (2015a, b) found it for 18 Latin American (LA) countries, Apergis and Ozturk (2015) for 14 Asian countries, You et al. (2015) for some selected countries, Shafiei and Salim (2014) for 29 OECD countries, Osabuohien et al. (2014) for 50 African countries, Culas (2012) for 9 LA countries, Shahbaz et al. (2012) for Pakistan, Hamit-Haggar (2012) for Canada, Jalil and Feridun (2011) for China, Pao and Tsai (2011) for BRIC countries, Leitão (2010) for 94 selected countries, Acaravci and Ozturk (2010) for Denmark and Italy out of 19 European countries and Zambrano-Monserrate et al. (2016) for Iceland. Again, several new studies have explored related issues using the EKC framework. For example, Li et al. (2021) found that economic growth and economic structure significantly affect carbon emissions positively and negatively respectively at the global level studying 147 countries. Wang et al. (2022a) find that the effects of official development assistance (ODA) on carbon emissions depend on the threshold level of urbanization. In a related study on 134 countries, Wang et al. (2022a) show that urbanization strengthens the positive correlation between the economy and carbon emissions and ecological footprint. Wang and Zhang (2020) studied BRICS countries and indicated that increasing research and development investment has a positive impact on decoupling economic growth from environmental pressure. Wang and Zhang (2021) show that trade openness has differential impacts on carbon emissions in high-income and low-income counties.

Likewise, many studies found no long-run inverted U-shaped relationship between GDP and environmental pollution. The studies of, for example, Kunnas and Myllyntaus (2007) for Finland^6^, Saidi and Mbarek (2017) for 19 emerging economies, Baek (2015) for 7 Arctic countries, Ghosh et al. (2014) for Bangladesh, Amin et al. (2012) for Bangladesh, Esteve and Tamarit (2012) for Spain, Dogan et al. (2020) for BRICST countries, Lin et al. (2016) for 5 African countries, Zhu et al. (2016) for 5 ASEAN countries, Zoundi (2017) for 25 African countries, Wang (2012) for 98 countries, Saleh et al. (2014) for Iran, Bagliani et al. (2008) for a set of 141 countries, Lise and Montfort (2007) for Turkey, Destek and Sinha (2020) for the OECD countries and Destek et al. (2018) have not confirmed the inverted U-shaped relationship between income and environmental degradation.

Some of the regional studies that have used different country clusters have confirmed validity for EKC some countries, but have failed to confirm the same for other countries included in the sample. For example, Urban and Nordensvärd (2018) studied the Nordic countries and observed EKC in Denmark, Iceland and Sweden but not in Norway and Finland in terms of total CO_2_ emissions. For other environmental degradation measures, the results were different (e.g. they confirmed the EKC relationship for Finland for per capita CO_2_ emissions). Ahmed et al. (2016) found EKC for 24 European countries for the long run, but not for the short run, and Nasir and Rehman (2011) for Pakistan in the short run. Apergis (2016) conducted a study on 15 OECD countries and found that the EKC hypothesis holds in 12 out of the 15 countries. Gormus and Aydin (2020) conducted research on top 10 innovative countries: Denmark, Finland, Germany, Israel, Korea, The Netherlands, Sweden, Switzerland, the UK, and the USA. However, the EKC hypothesis is valid for Israel, but not for the other countries.

### Studies confirming an N-shaped relationship

Conversely, there are arguments for the sacrifice of economic growth to protect the environment since economic growth may cause more environmental degradation (Coondoo and Dinda 2002). Likewise, many studies found that the initial U-shaped relationship would become N-shaped because there would be a new turning point at higher levels of growth resulting additional environmental degradation. Accordingly, in a study on Turkey, Akbostancı et al. (2009) concluded that there is no inverted U-shape relationship; rather, N-shape relationship is found between the income and emissions. Again, Azam et al. (2015) concludes that FDI inflows and real GDP significantly affect energy consumption . Studies have generally found the N-shaped EKC by including the cubic form of income in estimation models. The early studies of Grossman and Krueger (1995) and Panayotou (1997) have found an N-shaped relationship between economic development and sulfur dioxide (SO_2_). Moomaw and Unruh (1997) have found an N-shaped EKC for 16 countries which have undergone structural transition. Among other studies, Destek et al. (2020) validated an N-shaped EKC only for France among the G-7 countries and diversified shapes for the rest of the G-7 countries. Friedl and Getzner (2003) found it for Austria, Álvarez-Herranz and Balsalobre-Lorente (2015) for 28 OECD countries, Álvarez-Herranz et al. (2017) for 17 OECD countries, Poudel et al. (2009) for 15 LA countries, Ozturk and Acaravci (2013) for Turkey, Tiwari et al. (2013) for India and Allard et al. (2018) for 74 countries. Some of the studies, however, have ignored the possibility of an N-shaped EKC as they have not included the cubic term in estimations. Studies, for example, of Culas (2012), Zhang et al. (2016) and Liddle and Messinis (2015) fall into this category. Liddle and Messinis (2015), however, confirmed EKC for Hungary, Ireland, Italy, Japan, Luxembourg, the Netherlands, Belgium, Denmark, Finland, France, Norway, Portugal, Sweden and the USA out of 25 OECD countries.

### Studies on the Baltic countries

In the literature, the energy-growth nexus has been widely investigated over the last few decades. Several studies have been conducted on the direction of causality between energy consumption and economic growth focusing on the Baltic as well as the transition and post-transition European countries. However, there are still vivid debates, and the direction of such causality is not very clear in the literature.

The early study of Acaravci and Ozturk (2010) investigated the causality and long-run relationship between electricity consumption and economic growth in 15 transition countries, but could not confirm such relationship. Later, Sadorsky (2011) examined the impact of financial development on energy consumption in a sample of 9 Central and Eastern European frontier economies. The study found a positive and statistically significant relationship between financial development and energy consumption. However, the significance depends largely on the measures of financial development chosen. Chang et al. (2012) analysed the impact of the energy consumption and CO2 emissions ratio on both energy consumption and CO2 emissions in the Baltic countries. Focusing on the global financial crisis, they suggest that the crisis have hindered actions to combat CO2 emissions and excess consumption of energy. Again, Štreimikienė et al. (2012) assessed policies targeting energy intensity decrease in Lithuania in terms of efficiency, effectiveness and efficacy and to select the best policies able to increase energy efficiency in activities that are maintained. Roos et al. (2012) give an overview on the energy efficiency in the Baltic States, consumption of renewables and reduction of GHG emissions. Smiech and Papiez (2013) examined if any causal relationship between energy consumption and economic growth exists in nine CEE countries (Bulgaria, Czech Republic, Estonia, Hungary, Latvia, Lithuania, Poland, Romania and Slovakia) over 1995–2010 period. Their results indicate unresponsiveness of energy consumption to long-term equilibrium, while short-term relationship is bidirectional. They conclude that real gross fixed capital formation and labour force cause GDP growth. The study of Štreimikienė (2016) focuses on financial support from EU structural funds to sustainable energy development in Baltic States. Štreimikienė and Balezentis (2016) use the Kaya identity—that relates the greenhouse gas (GHG) emissions to energy use, economic growth and population growth—to analyse the feasibility of implementing the so-called EU 20–20–20 targets in the Baltic States^7^. They emphasized that policies to increase energy efficiency are the most important drivers of GHG emission reduction and achieving EU20–20–20 targets in Baltic States. Furuoka (2017) supported the conservation hypothesis while examining the connection between electricity consumption (renewable and non-renewable) and economic development in three Baltic countries, suggesting that economic development causes the expansion of renewable electricity consumption, but not vice versa.

Among the recent studies, Bui (2020) looked for possible transmission channels that allow financial development to affect environmental quality (CO_2_ emissions) using a global sample of 100 countries from 1990 to 2012 and confirmed positive and direct effect of financial development on environmental degradation. Ma and Fu (2020) investigated the influence of overall financial development and its components on energy consumption using data from a panel of 120 countries. Results of their study indicate that the overall financial development significantly positively impacts energy consumption worldwide and that the financial development also positively impacts energy consumption in developing countries but with no obvious effect in developed countries. Le (2020) evaluates the link between energy consumption and economic growth in 46 emerging market and developing economies (EMDEs) from 1990 to 2014. The findings demonstrate that energy usage, gross fixed capital formation, government expenditure, financial development and trade openness positively and significantly impact the economic growth. Results also imply that energy consumption and economic growth are interdependent, which forms a basis for policymakers to design effective energy and environmental policies. To analyse economic growth in Estonia, Latvia and Lithuania, Miskinis et al. (2021) discuss differences in development of the energy sector, among other main sectors, over the period 2000–2016. They found growing activities in the manufacturing and transport sectors to cause increase of final energy demand in these countries. Again, Janpolat et al. (2021) confirmed positive long-term relationship between financial development and energy use in 32 Belt and Road economies during 2000–2015, though no causal relationship between these variables was detected.

Thus, it is evident from the above discussion that fewer studies have been conducted on the validity of the EKC hypothesis in the Baltic region than studies directed towards the nexus between financial development and energy consumption, or on the energy–growth nexus. Also, we notice that the results on the EKC hypothesis are grossly inconclusive in the existing literature. This justifies further studies in this area.

## Data, model specification, and methodology

### Data

In the empirical analysis, this study employs annual data on per capita CO_2_ emissions, GDP per capita, energy consumption and financial development for the Baltic countries (Estonia, Latvia and Lithuania) over the period 1990-2018. These countries have been selected purposively as we wanted to concentrate only on the Baltic countries. But the selection of time span has been largely dictated by data availability for all the series, because observations on all variables and years were not available during the time of data collection in 2020. Thus, the dataset is an unbalanced panel of 3 countries followed over 29 years, and clearly the time dimension of the panel dataset ensures that the observations are for a long period of time. The data were sourced from the World Development Indicators (WDI) and the Financial Structure Database of the World Bank (available at http://data.worldbank.org). Initially, data were gathered up to 2019 for some variables. However, due to several incomplete data points, observations relating to the year 2019 were later excluded from the analysis. Among the variables, per capita CO_2_ emissions (measured in metric ton), real GDP per capita (measured in 2010 U.S. dollar) and energy consumption (measured in kg of oil equivalent per capita) have been collected from the WDI database, while data on ‘private credit by deposit money banks and other financial institutions percent of GDP’ were sourced from the Financial Structure Database. The variables have been transformed into natural logarithm to obtain the elasticities of the dependent variable (per capita CO_2_ emission) for the independent variables (real GDP per capita, the square of GDP, energy use financial development). Table 1 describes the variables used in the analysis with respective data sources. Notably, we tried to maximise the time span of the dataset as far as possible. Table 2 summarizes descriptive statistics for our data. Two of our main variables used in the exercise are CO_2_ emissions per capita and GDP per capita. As shown in Table 2, on average, the per capita CO_2_ emissions range between 1.26 metric tons (Latvia) and 2.54 metric tons (Estonia), while per capita GDP ranges between USD 9.192 (Latvia) and USD 9.453 (Estonia) during the sampling period. As respective figures for Lithuania are very close and lie between these values, the variations in terms of per capita CO_2_ emissions and per capita GDP are clearly minimal.

**Table 1:** Variable descriptions and sources of data

Variable name	Description	Data source
*Dependent variable*		
CO_2_ emissions	CO_2_ emissions (metric tons per capita)	World Development Indicators
*Independent variable*		
GDP per capita	Gross Domestic Product (constant 2010 US$)	World Development Indicators
Energy use	KG of oil equivalent per capita	World Development Indicators
Financial development	Private credit by deposit money banks and other financial institutions percent of GDP	Financial Structure Database

World Development Indicators (WDI) and Financial Structure Database have been compiled by the World Bank

**Table 2:** Summary statistics

Country	Variable	*N*	Mean	SD	Min	Max
Estonia	CO_2_ emissions	23	2.541	0.108	2.364	2.746
	GDP p.c.	26	9.453	0.347	8.816	9.901
	GDP p.c. squared	26	89.468	6.497	77.715	98.034
	Energy use	26	8.304	0.146	8.124	8.738
	Fin. development	25	3.775	0.674	2.238	4.630
Latvia	CO_2_ emissions	23	1.264	0.155	0.987	1.688
	GDP p.c.	25	9.192	0.381	8.545	9.697
	GDP p.c. squared	25	84.627	6.949	73.011	94.032
	Energy use	25	7.615	0.140	7.389	7.989
	Fin. development	24	3.448	0.820	1.902	4.549
Lithuania	CO_2_ emissions	23	1.461	0.114	1.246	1.789
	GDP p.c.	24	9.245	0.375	8.579	9.782
	GDP p.c. squared	24	85.596	6.887	73.593	95.684
	Energy use	25	7.882	0.184	7.620	8.434
	Fin. Development	24	3.224	0.627	2.312	4.062
Total	CO_2_ emissions	69	1.755	0.579	0.987	2.746
	GDP p.c.	75	9.299	0.380	8.545	9.901
	GDP p.c. squared	75	86.616	7.014	73.011	98.034
	Energy use	76	7.939	0.327	7.389	8.738
	Fin. Development	73	3.486	0.738	1.902	4.630

### Model specification

The study tests the existence of the EKC hypothesis by investing the effects of economic growth, energy consumption and financial development on CO_2_ emissions. Following previous literature (e.g. Ali et al. 2017; Begum et al. 2015), the empirical model is derived as a standard Cobb-Douglas production function. If constant returns to scale (CRTS) is assumed, the aggregate output at time t is given by

$${\mathrm{Y}}_{\mathrm{t}}=\mathrm{F}\left({\mathrm{K}}_{\mathrm{t}},{\mathrm{AL}}_{\mathrm{t}}\right)$$

where Y_t_ is GDP per capita, K_t_ is capital and AL_t_ represent the effective labour. Since human economic activities are the most important determining factor behind CO_2_ emissions, CO_2_ emissions can be expressed as a function of Y_t_ (i.e. GDP per capita):

$${\mathrm{CO}}_{2}\left(\mathrm{t}\right)=\upnu \left(\mathrm{F}\left(\mathrm{Yt}\right)\right)$$

Here, CO_2_ emission is directly linked with Y_t_ by the factor ‘ν’, which is the rate at which the production activities emit CO_2_. Not all forms of capital, however, are responsible for CO_2_ emissions. To be specific, consumption of coal, gas and electricity used in the production process is responsible for CO_2_ emissions. Therefore, the total capital can be described as a combination of CO_2_-emitting capital (K_e_) and non-CO_2_-emitting capital (K_n_):

$$\mathrm{K}={\mathrm{K}}_{\mathrm{e}}+{\mathrm{K}}_{\mathrm{n}}$$

Thus, we can rewrite the CO_2_ emissions function as

$${\mathrm{CO}}_{2}=\mathrm{\varnothing }{\mathrm{K}}_{\mathrm{e}}\left(\mathrm{Y}\right)$$

As mentioned above, we intend to describe environmental pollution as a function of real GDP, the square of real GDP, energy consumption and financial development in this study in order to investigate the validity of the EKC hypothesis in Baltic countries^8^. Hence, the empirical model in panel data format can be written as follows:

1 $${\mathrm{lnCO}}_{2\mathrm{it}}={\upbeta }_{0}+{\upbeta }_{1}{\mathrm{lnY}}_{\mathrm{it}}+{\upbeta }_{2}{\mathrm{lnY}}_{\mathrm{it}}^{2}+{\upbeta }_{3}{\mathrm{lnEC}}_{\mathrm{it}}+{\upbeta }_{4}{\mathrm{lnFD}}_{\mathrm{it}}+{\upmu }_{\mathrm{it}}$$

In the literature, these models are generally used to represent an IPAT approach as well (that is, Influence = Population, Affluence, and Technology). The IPAT approach was originally introduced by Ehrlich and Holdren (1971) but later reformulated by Rosa and Dietz (1998) to analyse the ‘Stochastic Impacts by Regression on Population, Affluence, and Technology’ called the STIRPAT. This is so named because model variables in Eq. (1) are widely used in the STIRPAT estimations (York et al. 2003; Fan et al. 2006; Lin et al. 2009).

In Eq. (1) above, t, i and μ refer to the time period, cross-section unit (here, country) and the residual term respectively. The term ‘ln’ stands for natural logarithm. CO_2_ refers to carbon dioxide (a greenhouse gas) emissions measured in metric tons per capita used as a proxy for environmental degradation, Y stands for gross domestic product (GDP) per capita (measured in millions of constant 2010 US dollars) to indicate income, and Y^2^ is the squared term of Y which is basically used to check the existence of the EKC. Checking the validity of the EKC will confirm whether economic growth is at the cost of environmental quality or not^9^. To examine the significance of other variables that might affect environmental quality, we include EC and FD. Here, EC and FD stand for energy consumption (measured in kilogram of oil equivalent per capita) and financial development (measured by ‘private credit by deposit money banks and other financial institutions percent of GDP’) respectively. To the best of our knowledge, use of this proxy for financial development is new as previous studies have generally used ‘private credit by deposit money banks percent of GDP’, or other close alternatives. Variables FD and EC have been included because they are linked with human activities and directly or indirectly affect the CO_2_ concentrations in the environment. For instance, financial development may attract foreign direct investment (FDI) to a country. This increases the pace of economic growth (Frankel and Romer 1999) as well as the environmental performance dynamics. Also, financial sector development may lead to the adoption of environmentally friendly and cleaner technologies to improve the global environment (Birdsall and Wheeler 1993; Frankel and Rose 2005). Besides, financial development may help attaining economic growth that may also lead to environmental degradation and industrial pollution. Again, previous studies have shown that consumption of renewable and non-renewable energy may have a reducing or an increasing effect on environmental pollution. For that reason, this study also includes energy consumption as an explanatory variable. In order to select the variables, the study particularly follows the works of Baek (2015) conducted on Arctic countries, Ali et al. (2017) conducted on Malaysia, Gormus and Aydin (2020) conducted on top 10 innovative countries, Allard et al. (2018) conducted on 74 selected countries, Shujah-ur-Rahman et al. (2019) conducted on Pakistan and Destek and Sarkodie (2019) conducted on 11 newly industrialized countries.

Here, the coefficient β_0_ measures the average environmental pressure when income has no influence on environmental degradation at all, while μ_it_ is the idiosyncratic error term. The coefficients β_1_, β_2_, β_3_ and β_4_ denote the direction and importance of the right-hand-side explanatory variables included in the model as noted above. Álvarez-Herranz and Balsalobre-Lorente (2016) and Allard et al. (2018) suggest that the EKC will adopt different shapes as follows^10^:

(i) β_1_ = β_2_ = 0 ⇒ Either no link between environmental degradation and income or a flat pattern
(ii) β_1_ > 0 and β_2_ = 0 ⇒ Environmental degradation increases with income monotonically
(iii) β_1_ < 0 and β_2_ = 0 ⇒ Environmental degradation increases with income monotonically
(iv) β_1_ > 0 and β_2_ < 0 ⇒ The classical inverted U-shaped EKC
(v) β_1_ < 0 and β_2_ > 0 ⇒ A U-shaped relation between environmental degradation and income

Our a priori expectations on the estimated coefficients are as follows. Consistent with the EKC hypothesis, the expected sign of β_1_ should be positive (greater than 0) because CO_2_ emission is positively related to economic growth of the country. The expected sign of β_3_ can be positive as well as negative depending upon the maturity level of the financial sector of the country. Arguably, leading investors and agencies may not care much about the environment if they are solely profit oriented. Therefore, financial development may increase environmental pollution (Ali et al. 2017). Also, it might be the case that a mature financial sector allocates funds only to the environmentally friendly projects and encourages the use of advanced technology. This may result in decreased emissions of energy pollutants (Ali et al. 2017). As more consumption of energy resources increases economic activities and boost CO_2_ emissions, β_4_ will have a positive value.

This study primarily uses the augmented mean group (AMG) estimator developed by Eberhardt and Bond (2009), Eberhardt and Teal (2010) and Bond and Eberhardt (2013). One of the major advantages of using this estimator is that it well considers, in econometric sense, the cross-sectional dependence and country-specific heterogeneity among countries. The other advantage of using this methodology is that it allows the examination of the parameters of non-stationary variables. Therefore, any pre-testing procedure (unit root or cointegration) is not required to use this approach (Destek and Sarkodie 2019). To start with the testing procedure, following Destek and Sarkodie (2019), the main panel data model expressed in Eq. (1) is estimated using its first-differenced form with T-1 year dummies in the following fashion:

2 $${\mathrm{\Delta CO}}_{2\mathrm{it}}={\upgamma }_{1}{\mathrm{\Delta Y}}_{\mathrm{it}}+{\upgamma }_{2}{\mathrm{\Delta Y}}_{\mathrm{it}}^{2}+{\upgamma }_{3}{\mathrm{\Delta EC}}_{\mathrm{it}}+{\upgamma }_{4}{\mathrm{\Delta FD}}_{\mathrm{it}}+{\sum }_{t=2}^{T}{p}_{t}\left({\mathrm{\Delta D}}_{\mathrm{t}}\right)+{\upmu }_{\mathrm{it}}$$

where ΔDt is first differences of T-1 year dummies and p_t_ is the parameters of year dummies. The coefficients on the (differenced) year dummies are then collected, which are referred to as ‘common dynamic process’. In the next step, estimated p_t_ parameters are transformed to φ_t_ variable which indicates a ‘common dynamic process’ as follows:

3 $${\mathrm{\Delta CO}}_{2\mathrm{it}}={\upgamma }_{1}{\mathrm{\Delta Y}}_{\mathrm{it}}+{\upgamma }_{2}{\mathrm{\Delta Y}}_{\mathrm{it}}^{2}+{\upgamma }_{3}{\mathrm{\Delta EC}}_{\mathrm{it}}+{\upgamma }_{4}{\mathrm{\Delta FD}}_{\mathrm{it}}+{\mathrm{d}}_{\mathrm{i}}\left({\mathrm{\varphi }}_{\mathrm{t}}\right)+{\upmu }_{\mathrm{it}}$$

4 $${\mathrm{\Delta CO}}_{2\mathrm{it}}-{\mathrm{\varphi }}_{\mathrm{t}}={\upgamma }_{1}{\mathrm{\Delta Y}}_{\mathrm{it}}+{\upgamma }_{2}{\mathrm{\Delta Y}}_{\mathrm{it}}^{2}+{\upgamma }_{3}{\mathrm{\Delta EC}}_{\mathrm{it}}+{\upgamma }_{4}{\mathrm{\Delta FD}}_{\mathrm{it}}+{\upmu }_{\mathrm{it}}$$

As the above equations show, therefore, firstly the group-specific regression model is adapted with φ_t_, and then the mean values of group-specific model parameters are computed (Destek and Sarkodie 2019). For example, we may compute the parameter of economic growth (γ_1_) as ${\gamma }_{1, AMG}= \frac{1}{N} \sum_{i=1}^{N}{\gamma }_{1,i}$.

In addition, the study also utilizes the two other panel time-series estimators, allowing for heterogeneous slope coefficients across group members: the Pesaran and Smith (1995) mean group (MG) estimator and the Pesaran (2004) common correlated effects mean group (CCEMG) estimator. Pesaran (2001) shows that consistent estimates of the regression (slope) coefficients can be obtained by the CCEMG panel data estimators without the need to determine the number of unobserved common factors, given that the regressors are stationary and exogenous. Consistency of the estimators is robust to non-stationarity or common factors (Pesaran 2001), cointegration between them (Kapitanios et al. 2011) or spatial correlation in the idiosyncratic errors (Pesaran and Tosetti 2011)^11^. For checking robustness of the results, the study also uses the random coefficients model developed by Swamy (1970), which does not impose the assumption of constant parameters across panels.

### Methodology

#### Cross-sectional dependence

It is not a surprise to see that economic, financial and environmental indicators of different countries represent a high degree of correlation, especially if countries are clustered from any specific region. Intuitively, there might be the effect of some unobserved common factors in a sample of cross-section units, which are common to all units and affect each of them although possibly in different ways. For this reason, this study first examines the existence of cross-sectional dependence among countries using the CD test described in Pesaran (2004) and Pesaran (2015). The test is all about an investigation of the mean correlation between panel units. The null hypothesis is either strict cross-sectional independence (Pesaran 2004) or weak cross-sectional dependence (Pesaran 2015).

Following Gormus and Aydin (2020), the test statistics proposed by Breusch and Pagan (1980) were used for testing the cross-sectional dependence for Eq. (1) as follows:

5 $$\mathrm{CD}=T\sum\nolimits_{i=1}^{N-1}\sum\nolimits_{j=i+2}^{N}{\widehat{\rho }}_{ij}^{2}$$

where ${\widehat{\rho }}_{ij}^{2}$ shows the correlation between errors calculated from Eq. (1). However, the test statistic calculated in Eq. (3) may provide different results in large samples. For that reason, Pesaran (2004) proposed a test statistic as follows:

6 $${\mathrm{CD}}_{\mathrm{LM}1}=\sqrt{\frac{1}{N(N-1)}}\sum\nolimits_{i=1}^{N-1}\sum\nolimits_{j=i+1}^{N} (T{\widehat{\rho }}_{ij}^{2}-1)$$

To get the above test statistic, we need to make some corrections to the test statistic given in Eq. (3). This allows us to test the cross-section dependence in large samples. However, where the observation (N) is greater than the time (T), Eq. (4) is regulated by Pesaran (2004), and the following test statistic is obtained:

7 $${\mathrm{CD}}_{\mathrm{LM}2}=\sqrt{\frac{2T}{N\left(N-1\right)}}(\sum\nolimits_{i=1}^{N-1}\sum\nolimits_{j=i+1}^{N}{\widehat{\rho }}_{ij})$$

For all three test statistics noted above, the null hypothesis shows that there is no cross-sectional dependence, while the alternative hypothesis refers to cross-sectional dependence.

#### Slope homogeneity

In addition, slope homogeneity is examined in the study with ${\Delta }_{\sim }$ and ${\Delta }_{\sim }$ adj tests proposed by Pesaran and Yamagata (2008). The test statistic of the delta tests is obtained as follows (Gormus and Aydin 2020):

8 $$\widehat{\Delta }=\sqrt{N}\left(\frac{{N}^{-1} \tilde{S }-k}{\sqrt{2k}}\right)$$

This is an extended version of the method proposed by Swamy (1970) for random coefficient regression model. Here, $\tilde{S } s$ shows the modified Swamy statistics. Delta test statistics have been modified (${\widehat{\Delta }}_{adj}) by$ Pesaran and Yamagata (2008) that examine slope homogeneity under the assumption of normally distributed errors. For both test statistics, the null hypothesis shows homogeneity, while the alternative hypothesis represents the heterogeneous slope.

#### Unit root and cointegration tests

Following the analyses of Destek and Sinha (2020), the study considers the cross-sectional dependence; we used Pesaran’s (2007) cross-sectional ADF (CADF) unit root test. The empirical form of the test is described below:

9 $$\Delta {y}_{it}={\alpha }_{i}+{\rho }_{i}{y}_{it-1}+ {\beta }_{i}{\overline{y} }_{t-1}+ \sum\nolimits_{j=0}^{k}{\gamma }_{ij}\Delta {\overline{y} }_{it-1}+\sum\nolimits_{j=0}^{k}{\delta }_{ij}{y}_{it-1}+{\epsilon }_{it}$$

where αi is constant, k is the lag specification and y_t_ is the temporally defined cross-sectional average. Then, t-statistics are acquired from distinct ADF statistics. Besides, cross-sectional IPS (CIPS) is attained from the mean of CADF values for individual cross-sections:

10 $$CIPS=\left(\frac{1}{N}\right)\sum\nolimits_{i=1}^{N}{t}_{i} (N, T)$$

To analyse the long-run association among the model parameters, we utilized ECM-based cointegration technique developed by Westerlund (2007). In the estimation process, Gt, Ga, Pt and Pa are computed to test the cointegrating association among the model parameters. Statistical significance of the error correction term in the restricted VECM is used to calculate the statistics. The estimation model can be formed as per the following:

11 $$\Delta Y_{it}=\delta'_id_t+\alpha_iY_{i,t-1}+\lambda'_iX_{i,t-1}+\sum\nolimits_{j=1}^{p_i}\alpha_{ij}\Delta Y_{i,t-j}+\sum\nolimits_{j=-q_i}^{p_i}\gamma_{ij}\Delta X_{i,t-j}+\mu_{it}$$

where d_t_ indicates the constant term, such that

$$d_t=\left\{\begin{array}{l}1,\;constant\;trend\\0,\;no\;constant\;trend\\\left(1,t\right)',\;constant\;trend\end{array}\right.$$

Furthermore, ai determines the speed of adjustment.

#### Heterogeneous panel causality

To examine the causal connections between variables, this study uses heterogeneous panel causality tests of Dumitrescu and Hurlin (2012). This methodology is a modified version of Granger causality and adapted to heterogeneous panel data. In addition, the Monte Carlo simulations show that this methodology gives consistent results under cross-sectional dependency. Dumitrescu and Hurlin (2012) define the average statistic ${W}_{N,T}^{Hnc}$ associated with the null homogeneous non-causality (HNC) hypothesis as

12 $${W}_{N,T}^{Hnc}=\frac{1}{N} \sum\nolimits_{i=1}^{N}{W}_{i,T}$$

where ${W}_{N,T}^{Hnc}$ statistic is obtained with averaging each Wald statistics for cross-sections and *W*_*i,T*_ denotes the individual Wald statistics for the i’th cross-section unit corresponding to the individual test H_0_: β_i_ = 0. Here, the average statistic ${W}_{N,T}^{Hnc}$ sequentially converges in distribution as follows:

13 $${Z}_{N,T}^{Hnc}=\sqrt{\frac{N}{2K}} \left({W}_{N,T}^{Hnc}-K\right)\to N \left(0, 1\right)$$

with ${W}_{N,T}^{Hnc}=\frac{1}{N} \sum_{i=1}^{N}{W}_{i,T}$ if T→∞ first and then N→∞. The null hypothesis (H_0_) of ‘there is no homogeneous causality’ has been tested against the alternative hypothesis (H_1_) that ‘the causal relationships are heterogeneous’ all along the testing procedure.

## Empirical findings and discussions

The empirical findings on the CD tests, panel unit roots tests, cointegration tests (Pedroni-ADF, Pedroni-PP and Kao-ADF tests), long-run panel estimations and causality tests have been discussed in this section. In the first step of the analysis, the cross-sectional dependence and country-specific slope heterogeneity were examined. This study utilizes Pesaran (2004), Pesaran (2015) and Breusch-Pagan LM tests (Breusch and Pagan 1980) to check the cross-sectional dependence. The results are presented in Tables 3 and 4. We see that the null hypothesis of no cross-sectional dependence among countries is rejected for all tests, which confirm that the cross-sections of the data are interdependent. Intuitively, this means that if a shock occurs in one of the selected countries, then it may transmit to other countries. The CD test is also undertaken to investigate the cross-sectional dependence in the dynamic panels for the residuals (Table 5). Results of all three tests in this connection reject the null hypothesis and confirm cross-sectional dependence in the data. Next, in the second step, slope heterogeneity tests suggested by Pesaran and Yamagata (2008), Pesaran (2015) and Blomquist and Westerlund (2013) have been conducted to validate the CD test results. The results of the slope homogeneity test (Table 6) have all been rejected at 5% level which confirm the presence of a country-specific slope heterogeneity among the selected countries.

**Table 3:** Pesaran (2015) pre-estimation test on cross-section correlation (CD Test)

Variable	CD-test	*P* value	Av. Joint T	Mean ρ	Mean abs(ρ)
CO_2_ emissions	6.388	0.000	23.00	0.77	0.77
GDP p.c.	8.465	0.000	24.33	0.99	0.99
GDP p.c. squared	8.463	0.000	24.33	0.99	0.99
Energy use	7.158	0.000	25.00	0.83	0.83
Fin. Development	7.562	0.000	24.00	0.89	0.89

CD presents the Pesaran (2004) cross-section dependence statistic. Under the null hypothesis of cross-section independence, CD ~ N(0,1). The average and absolute correlation coefficients have been reported. *P* values close to zero indicate data are correlated across panel groups. Panels with missing values have been used

**Table 4:** Bias-adjusted LM test of error cross-section independence

Test	Statistic	*P* value
Breusch-Pagan LM	36.59	0.0000
Bias-adjusted LM (LM adj*)	32.49	0.0000
Pesaran CD (LM CD*)	5.906	0.0000

^*^Two-sided test

**Table 5:** Cross-sectional dependence (CD) test results (for residuals)

Test	Statistic
Pesaran CD test	2.454**
Friedman test	55.853***
Frees test	-0.012***

Asterisks (*, ** and ***) indicate statistical significance at 10, 5 and 1% level respectively

**Table 6:** Pesaran and Yamagata (2008) slope homogeneity (i.e. PY 2008) (panels with missing values) and Blomquist and Westerlund (2013) slope heterogeneity (i.e. BW 2013) (balanced panel) test results

Statistic (for homogeneity)	PY 2008 test		BW 2013 test	
	Value	*P* value	Value	*P* value
Delta ($\stackrel{\sim }{\Delta }$)	-2.379*	0.017	-2.640**	0.008
Delta adjusted (${\stackrel{\sim }{\Delta }}_{adj.}$)	-2.950**	0.003	-3.068**	0.002

Tests are performed to reduce small-sample bias in HAC estimation (see Andrews and Monahan 1992). Double asterisks (**) indicate significance at 5% level

In the third step, these CD test results are used to employ an appropriate panel unit root test. Accordingly, CADF (Cross-sectional Augmented Dickey Fuller) and CIPS (Cross-sectional Im, Pesaran, and Shin) unit root tests have been employed. These tests allow for cross-sectional dependence in the data while assessing the order of integration level of the model parameters. The results of these tests are presented in Table 7 which affirm that all variables are non-stationary at level, but they become stationary at first differenced form.

**Table 7:** CADF (Cross-sectional Augmented Dickey Fuller) and CIPS (Cross-sectional Im, Pesaran, and Shin) panel unit root test results

Variable	CADF		CIPS	
	Z[t-bar]	P-value	Z[t-bar]	CV (1%)
Case 1: level form				
*Deterministics chosen (models with): constant only*				
CO_2_ emissions	-0.861	0.195	-2.599	-2.51
Energy use	-0.196	0.422	-1.916	-2.51
Electricity consumption	-4.340	0.000	-3.101	-2.51
Renewable energy use	-1.688	0.046	-2.907	-2.51
Energy intensity	-3.175	0.001	-2.584	-2.51
GDP per capita	0.275	0.608	-2.233	-2.51
GDP per capita squared	0.335	0.631	-2.195	-2.51
Financial development	-0.287	0.387	-1.202	-2.51
*Deterministics chosen (models with): constant and trend*				
CO_2_ emissions	-1.112	0.133	-3.183	-3.30
Energy use	-2.147	0.016	-3.358	-3.30
Electricity consumption	-2.107	0.018	-4.016	-3.30
Renewable energy use	-4.632	0.000	-3.715	-3.30
Energy intensity	-1.811	0.035	-2.772	-3.30
GDP per capita	2.031	0.979	-2.333	-3.30
GDP per capita squared	1.982	0.976	-2.375	-3.30
Financial development	1.094	0.863	-1.368	-3.30
Case 2: first difference				
*Deterministics chosen (models with): constant only*				
CO_2_ emissions	-6.286	0.000	-5.361	-2.44
Energy use	-5.778	0.000	-5.648	-2.44
Electricity consumption	-5.113	0.000	-5.763	-2.44
Renewable energy use	-5.834	0.000	-5.361	-2.44
Energy intensity	-5.338	0.000	-5.423	-2.44
GDP per capita^#^	-1.378	0.084	-5.075	-2.44
GDP per capita squared^#^	-1.371	0.085	-5.010	-2.44
Financial development^#^	-1.430	0.076	-3.651	-2.51
*Deterministics chosen (models with): constant and trend*				
CO_2_ emissions	-5.407	0.000	-5.379	-3.30
Energy use	-5.260	0.000	-5.874	-3.10
Electricity consumption	-4.296	0.000	-5.898	-3.10
Renewable energy use	-4.774	0.000	-5.376	-3.10
Energy intensity	-5.260	0.000	-5.449	-3.10
GDP per capita^#^	-1.290	0.099	-5.595	-3.30
GDP per capita squared^#^	-1.343	0.090	-5.609	-3.30
Financial development^#^	-3.852	0.000	-3.927	-3.30

All variables have been transformed into natural logs. For CADF tests, cross-sectional averages in the first period are extracted, and extreme t-values have been truncated. However, results do not differ significantly even if no truncation of extreme t-values is made (‘trunoff’ option is used with trend). Following Burret et al. (2016) and Hoechle (2007), we select the ideal lag length by using Newey and West’s (1994) plug-in procedure at (4 ∗ (T / 100)2/9 = 1.09751293089 ≈ 1)

^##^For second difference, respective *P* values equal 0.000. Similar *P*-values have been used in Destek et al. (2018)

Having identified variables with the unit root processes, the study then (in the fourth step) aims to investigate if any long-run relationship is present among the variables. Accordingly, the study utilizes the cointegration test of Westerlund (2007), which is a second-generation panel cointegration test that accounts for cross-section dependence between cross-section units (or panel groups). When the results of Westerlund’s cointegration test are evaluated (Table 8), Ga and Pa statistics confirm the null hypothesis, whereas Gt and Pt statistics strongly reject it. Thus, it can be inferred that the model parameters are cointegrated to have long-run relationships. Additionally, Pedroni (1999) and Kao (1999) cointegration tests have also been employed. According to the results, both of the cointegration tests reject the null hypothesis of no cointegration between variables. Similarly, Kao cointegration test results reject the null hypothesis (Table 9).

**Table 8:** Westerlund panel cointegration test results

Statistic	Value	Z-value	*P* value	Robust *P* value
Gt	-5.265	-4.584	0.000	0.010
Ga	-4.111	2.655	0.996	0.860
Pt	-7.629	-3.081	0.001	0.010
Pa	-4.425	1.904	0.972	0.700

Results for H0: no cointegration. Bootstrapping critical values have been used under the null hypothesis. These results have been used following previous studies (see, e.g. Destek and Sinha 2020; Destek et al. 2018; Baloch et al. 2020; Rauf et al. 2018)

**Table 9:** Other panel cointegration test results

Cointegration tests	Statistic	*P* value
Pedroni-ADF	-4.099	0.000
Pedroni-PP	-3.656	0.000
KAO-ADF	-2.751	0.000

In the fifth step, the effects of economic growth (measured by real GDP per capita), squared values of economic growth, energy consumption and financial development on CO_2_ emissions have been investigated with mean group (MG), augmented mean group (AMG) and common correlated effects mean group (CCEMG) estimators. According to the MG estimation results (presented in Table 10), the coefficient of real GDP per capita is negative and statistically significant (at 1% level) in all three countries. Again, the coefficient of the square of real GDP per capita is positive and statistically significant at 1% level in all three included countries as well. Therefore, a U-shaped relationship between economic growth and environmental degradation exist in all three countries which confirms the invalidity of EKC hypothesis. This segment of the findings resonates with the results obtained by, for example, Destek and Sinha (2020) for renewable and non-renewable energy consumption in the OECD countries. However, this finding contradicts the findings of Ulucak and Bilgili (2018), Sarkodie and Strezov (2018) and many others. Contrary to EKC hypothesis, a U-shaped EKC essentially tells us about reduced energy efficiency. This can happen because of using outdated technologies, for instance, in the energy sector that greatly influence economic productivity. This also demonstrates a situation where energy intensity increases per unit of economic outcome and, consequently, energy efficiency reduces (Sarkodie and Strezov 2019a, b). These results also represent that economic growth sets off environmental degradation after a threshold. But economic growth may lead the degradation to decrease at earlier process of its trend path till that threshold (Destek et al. 2018). Moreover, as the countries in the Baltic region are relatively technologically advanced, the pollution haven hypothesis may also have influenced the shape of the EKC in this country. According to this hypothesis, firms in these countries may have tried to avoid the costs of strict environmental regulations (and high energy prices) by locating productions in countries with laxer environmental laws. Thus, environmental degradation declines initially. These arguments are similar to those provided by, for instance, Dinda (2004) and Sarkodie and Strezov (2019a, b). In AMG and CCEMG model estimations, the coefficients of the economic growth and squared economic growth variables which have negative and positive signs are required for a U-shaped relationship. However, except AMG estimations for Latvia, these coefficients are not statistically significant at any conventional level, even though such relationships support a U-shaped relationship invalidating the EKC hypothesis.

**Table 10:** Mean group (MG), augmented mean group (AMG) and common correlated effects mean group (CCEMG) estimation results

	Constant	GDP	GDP^2^	Energy use	Fin. Dev.
Estonia					
MG	39.702** (14.628)	-8.946** (3.038)	0.488** (0.159)	0.480*** (0.080)	-0.047 (0.056)
AMG	3.966 (10.134)	-1.323 (2.120)	0.089 (0.111)	0.418*** (0.048)	-0.034 (0.033)
CCEMG	-29.869* (15.164)	4.434 (10.585)	-0.252 (0.562)	0.560*** (0.114)	-0.035 (0.066)
Latvia					
MG	57.851*** (11.189)	-13.338*** (2.328)	0.710*** (0.126)	0.705*** (0.099)	0.144*** (0.033)
AMG	24.086** (7.444)	-5.857*** (1.584)	0.311*** (0.085)	0.583*** (0.056)	0.110*** (0.018)
CCEMG	-1.747 (11.348)	-20.560 (15.893)	1.159 (0.854)	0.446 (0.246)	0.099 (0.092)
Lithuania					
MG	48.987*** (7.928)	-11.018*** (1.710)	0.592*** (0.093)	0.407*** (0.071)	0.137** (0.043)
AMG	7.910 (6.544)	-2.098 (1.417)	0.118 (0.076)	0.356*** (0.037)	0.070** (0.024)
CCEMG	8.130 (29.009)	0.783 (23.469)	-0.040 (1.241)	0.277 (0.190)	-0.021 (0.151)
Panel					
MG	48.853*** (5.653)	-11.096*** (1.377)	0.596*** (0.070)	0.518*** (0.129)	0.140*** (0.004)
AMG	10.039 (11.024)	-2.324 (2.520)	0.103*** (0.017)	0.446*** (0.090)	0.052 (0.056)
CCEMG	-7.829 (11.383)	-5.114 (7.794)	0.289 (0.439)	0.428*** (0.082)	0.014 (0.043)

All variables have been transformed into natural logs. Asterisks (*, ** and ***) indicate statistical significance at 10, 5 and 1% level respectively. Numbers in parentheses are the standard errors

in the above table, positive and highly significant coefficients of the energy use variable (in all three countries and in almost all regressions) confirm that an increase in energy consumption leads to an increase in environmental degradation in all three countries. According to the results of panel regression, an increase in energy use by 1% will increase CO_2_ emissions by 0.518%. This result also means that while clean and renewable energy technologies may promote a clean environment—which is not discussed in this study—fossil fuel energy technologies increase environmental pollution. Thus, increased use of fossil fuel simply adds to high level of CO_2_ emissions in these countries. These results are in agreement with, for instance, Sarkodie and Adams (2018). The coefficients of the financial development variable are statistically significant in the panel regression as well as in case of Latvia and Lithuania. According to the results of panel regression, for example, an increase in financial development by 1% will increase CO_2_ emissions by 0.140% in the Baltic countries. This type of results essentially supports the hypothesis that financial development leads to higher emission rates and, therefore, has clearly and significantly affected the environmental degradation of these countries. For Estonia, however, no such link can be established based on these data. That is, the country’s financial improvement may not have any clear impacts on environmental degradation. Both positive and negative linkages between financial development and environmental degradation have been noted in the literature. Similar results have been found in several studies before [see, e.g. Zhang 2011 (for China); Boutabba 2014; Khan et al. 2018 (for India); Shahbaz et al. 2016 (for Pakistan); Bekhet et al. 2017 (for the Gulf Cooperation Council (GCC) countries; Al-Mulali et al. 2015a, b (for 23 countries); and Jiang and Ma 2019 (for global sample of countries)]. Finally, in group panel MG estimations, the study finds that the coefficients of GDP per capita and square of GDP per capita are highly significant statistically with negative and positive signs respectively. Again, the coefficient of GDP per capita is negative insignificant, but the coefficient of the squared value is positive and statistically significant. Therefore, we can confirm a U-shaped relation between economic growth and environmental degradation supporting the EKC hypothesis in the Baltic countries, if the countries are taken as a single region. We see, however, that the coefficient of the financial development variable is positive and highly significant. Therefore, financial development affects environmental degradation directly in the Baltic region as a whole.

In the sixth and final step of the analysis, the causal relationships between CO_2_ emissions, economic growth, energy consumption and financial development have been examined using the heterogeneous panel causality method. The results, as shown in Table 11, demonstrate bidirectional causalities between CO_2_ emissions and economic growth, CO_2_ emissions and energy use, CO_2_ emissions and financial development, energy use and economic growth as well as between energy use and financial development. The results, however, reveal a unidirectional causality between economic growth and financial development. The causal link between economic growth and energy use can be justified as follows. High level of income causes more natural resource extraction in the Baltic economies, which reduces the biocapacity of the environment and raises CO_2_ emissions. The bidirectional causality between CO_2_ emissions and energy uses, economic growth and financial development, CO_2_ emissions and financial development and between energy use and economic growth are self-explanatory as one simply feeds the other. A unidirectional causality from economic growth to financial development suggests that as the economy grows in terms of per capita, GDP financial development also improves, but not the vice versa. One plausible explanation to this result might be that although the Baltic countries tend to depend less on conventional energy sources like fossil fuels, consumption of energy per capita is still very high in these countries. Moderately high heating costs due to the cold climate, greater need for individual transportation and high levels of income in general are considered the main factors behind this high level of energy demand. Statistically significant Wald statistics accordingly confirm that there is causal relationship between economic growth and energy use, meaning that income indeed will have direct effect on energy use. Hence, we may confirm the feedback hypothesis which suggests a bidirectional causal relationship between energy consumption and real GDP^12^.

**Table 11:** Heterogeneous panel Granger causality test results [Dumitrescu and Hurlin 2012 (DH 2012) and Karavias and Sarafidis, 2021 (KS 2021)]

Null hypothesis	DH 2012		KS 2021	
	Wald stat.	*P* value	Wald stat.	*P* value
lnCO_2_ ⇏ lnEUS	4.978***	0.000	57.123***	0.000
lnEUS ⇏ lnCO_2_	7.553***	0.000	1.674	0.433
lnCO_2_ ⇏ lnGDP^##^	7.104**	0.004	45.433***	0.000
lnGDP ⇏ lnCO_2_	7.067***	0.000	358.662***	0.000
lnCO_2_ ⇏ lnFD^##^	8.227***	0.000	59.503***	0.000
lnFD ⇏ lnCO_2_	4.377***	0.000	71.827***	0.000
lnEUS ⇏ lnGDP	6.615***	0.000	4379.814***	0.000
lnGDP ⇏ lnEUS	3.935***	0.000	19.284***	0.000
lnEUS ⇏ lnFD	6.528***	0.000	4954.773***	0.000
lnFD ⇏ lnEUS	3.070*	0.011	36.757***	0.000
lnGDP ⇏ lnFD	1.609	0.456	2.210	0.137
lnFD ⇏ lnGDP^#^	5.719***	0.001	1832.745***	0.000

For the Dumitrescu and Hurlin (2012), ⇏ means ‘does not Granger cause’. *P* values correspond to Z-bar *P* values obtained by using Stata’s ‘xtgcause’ routine. For the Karavias and Sarafidis (2021), however, the alternative hypothesis is the left-hand-side variable does Granger-cause the right-hand-side variable for at least one panel variable. This test uses dynamic model with lag length selection (up to 4 lags) based on BIC, with cross-sectional heteroskedasticity-robust standard errors. For the Karavias and Sarafidis (2021), Stata’s ‘xtgranger’ routine is used. *, ** and *** indicate statistical significance at 10, 5 and 1% level, respectively. ^#^The second lag is used. ^##^The third lag is used

### Robustness analysis

Next, we conducted two robustness checks to verify the reliability of the results. First, to validate the robustness of the mean group estimation outcomes, this study employs the random coefficients modelling approach to take care of slope heterogeneity and re-estimated the models. Second, the study re-estimated the models using a balanced panel dataset for a shorter sample period (1993–2014). The results are provided in Table 12. This dataset was created from the original unbalanced dataset for the sample period of 1990–2018. The corresponding results of the random coefficients estimations have been presented in Table 13. Results corroborate with the findings from the MG, AMG and CCEMG analyses regarding the predicted signs as well as the elasticity estimates. The study also performs robustness analysis for checking the causality among the variables. So, the study tests for Granger non-causality in heterogeneous panel data models using the methodology developed by Juodis et al. (2021). Results of such analysis remain largely unperturbed. Hence, the robustness of the findings across alternative estimation techniques is affirmed^13^.

**Table 12:** Random coefficients (RC) model estimation results

	Constant	GDP	GDP^2^	Energy use	Fin. Dev.
Estonia	45.512*** (6.780)	-10.160*** (1.491)	0.551*** (0.077)	0.473*** (0.080)	-0.029 (0.052)
Latvia	57.253*** (6.162)	-13.179*** (1.348)	0.702*** (0.072)	0.689*** (0.070)	0.144*** (0.033)
Lithuania	46.404*** (6.579)	-10.509*** (1.464)	0.565*** (0.079)	0.436*** (0.077)	0.131** (0.045)
Panel	49.100*** (7.068)	-11.156*** (1.609)	0.510*** (0.083)	0.530*** (0.100)	0.077 (0.066)

Asterisks (*, ** and ***) indicate statistical significance at 10, 5 and 1% level respectively. Numbers in parentheses are the standard errors

**Table 13:** AMG, CCEMG and random coefficients (RC) estimation results using smaller (balanced) sample: 1993–2014

	Constant	GDP	GDP^2^	Energy use	Fin. Dev.
Estonia					
AMG	-4.513 (7.447)	-0.195 (1.560)	0.020 (0.082)	0.899*** (0.089)	-0.046* (0.023)
CCEMG	-22.012 (14.251)	-6.158 (8.564)	0.328 (0.457)	1.108*** (0.150)	-0.084 (0.053)
RC	8.148 (10.016)	-3.126 (2.027)	0.171 (0.108)	1.072*** (0.117)	-0.059 (0.031)
Latvia					
AMG	10.090 (9.406)	-3.753 (1.935)	0.190 (0.106)	1.249*** (0.189)	0.072** (0.022)
CCEMG	-18.896 (12.026)	-20.560 (15.893)	1.159 (0.854)	0.446 (0.246)	0.099 (0.092)
RC	16.143 (12.444)	-5.465* (2.479)	0.276* (0.136)	1.541*** (0.209)	0.068* (0.027)
Lithuania					
AMG	14.847 (10.974)	-3.635 (2.430)	0.207 (0.133)	0.343*** (0.086)	0.051 (0.031)
CCEMG	-8.377 (34.499)	-2.592 (31.053)	0.174 (1.644)	0.930** (0.305)	0.127 (0.161)
RC	57.123*** (9.976)	-12.872*** (2.143)	0.702*** (0.117)	0.382* (0.156)	0.073 (0.039)
Panel					
AMG	7.580 (8.360)	-3.694*** (0.066)	0.198*** (0.009)	0.836** (0.299)	0.041 (0.069)
CCEMG	-17.155** (6.312)	-6.365** (2.456)	0.368* (0.152)	0.911*** (0.133)	0.035 (0.073)
RC	27.301 (17.055)	-7.205* (3.333)	0.386* (0.184)	1.003** (0.358)	0.025 (0.047)

Asterisks (*, ** and ***) indicate statistical significance at 10, 5 and 1% level respectively. Numbers in parentheses are the standard errors

## Conclusions and policy recommendations

The EKC hypothesis—an inverted U-shaped relationship between economic growth and environmental degradation—has been examined in this paper for three Baltic countries, namely Estonia, Latvia and Lithuania. The study accordingly verifies the EKC hypothesis for CO_2_ emissions using a linear function controlling for non-renewable energy use and financial development. For this purpose, the paper uses annual data covering 29 years (from 1990 to 2018). So, the diagnostic issues relevant for a macro panel (T > N) study like this were important to cope with. First, the applicability of second-generation panel data models is validated by the cross-sectional dependence test and slope heterogeneity test. Then unit root tests show that the variables are first-order integrated and cointegration tests reveal the long-run relationship among the variables. The study then employs several mean group estimators (e.g. MG, AMG and CCEMG) and heterogeneous panel causality method. These methods are suitable for panels with cross-sectional dependence and country-specific heterogeneity. Data aggregation bias may also result when we use a panel dataset clustering a number of countries. In order to address the bias, therefore, the paper also employs time series data at individual country levels while examining the EKC hypothesis.

According to the results of the econometric analyses, the inverted U-shaped EKC hypothesis is not validated by the panel of the Baltic countries. This clearly means the role of economic growth in reducing CO_2_ emissions is generally insignificant in this part of the world. In panel estimations, the study finds a U-shaped relationship between economic growth and CO_2_ emissions. Similarly, the study finds a U-shaped EKC for all three countries when the estimation results were evaluated for each of the countries individually. Such U-shaped association essentially suggests that economic growth is not vital in reducing CO_2_ emissions in the Baltic countries. In addition, the study finds that increased energy consumption leads to increased environmental degradation in this region and in all three Baltic countries when they are studies individually. Two separate heterogeneous panel causality tests were applied, and the results confirm bi-directional causality links between CO_2_ emissions and economic growth, CO_2_ emissions and energy use, CO_2_ emissions and financial development, energy use and economic growth and between energy use and financial development. The study also found unidirectional causality running from financial development to economic growth. The results obtained in this study reveal the impacts of economic growth and non-renewable energy consumption on CO_2_ emissions and are important for respective policymakers in the Baltic countries. Thus, the study contributes to the existing literature of environmental and energy economics, especially in attaining the goals of the 7th SDG.

Baltic countries are quite different in terms of how much they depend on fossil fuels. For instance, energy in Estonia, especially electricity production, is largely dependent on fossil fuels, whereas non-renewable energy sources provide almost half of the electricity used in Latvia. In case of Lithuania, however, renewable energy constituted only around one-quarter of overall electricity generation. Such country-level differences in fossil-fuel dependence might explain, at least to some extent, the non-existence of the EKC hypothesis in some of the Baltic countries though all of these three countries are now part of the European Union system. Again, based on the empirical findings of the study, non-renewable energy consumption increases the environmental degradation in these countries. Therefore, the Baltic countries are recommended to move away from fossil-fuel-dependent energy consumption growth policies to mitigate environmental degradation. It is now widely accepted that the share of renewable energy is indeed a determining factor in reducing CO_2_ emissions. Therefore, it is essential to try to expand renewable energy sources including, but not limited to, solar, wind, biofuel and geothermal energy.

The findings of this empirical exercise suggest some important policy implications. The non-existence of the EKC hypothesis indicates that environmental sustainability in the Baltic nations may not be provided with economic growth alone. In other words, the prevailing environmental regulation standards of the Baltic countries are inadequate for the purpose of reducing CO_2_ emissions. The policymakers in the Baltic countries should focus more on shifting towards renewable energy-based solutions from their current fossil fuel-based solutions. But this must not happen at the cost of harming their existing economic growth pattern. Environmental regulatory standards should be changed or reframed to achieve this objective. Renewable energy technologies can be improved, for instance, by introducing environmental taxes, eliminating subsidies in applicable cases and appropriately defining the public property rights. In this regard, phase-wise transition should be in place to accommodate both households and the industrial sectors (Destek and Sinha 2020). This way, these countries may internalize the negative externalities caused by the fossil fuel-based economic growth.

The study is obviously not without limitations. This suggests that future research should further investigate the relationship between economic growth and environmental degradation possibly by considering other possible shapes. For example, this study uses econometric tools in order to explain the patterns which have been already observed. Future studies should, therefore, incorporate an alternative research tool (e.g. qualitative research design). Also, perhaps a different functional form was required (including the cubic term perhaps) to explain things in a better way. It is also possible that the empirical model employed in this paper could not capture all the dimensions. Future empirical works could seek to incorporate other variables (e.g. corruption, governance and trade openness) that help define the differences between countries or cluster of countries. Future research might also aim to determine the turning points associated with an EKC which can explain whether environmental degradation may have permanent effects on the economy. We hope to address these issues in future research.

## Data Availability

The datasets used and/or analysed during the current study are available from the corresponding author on reasonable request.

## Footnotes

1 For further information on goal 7 of the UN’s Sustainable Development Goals, see https://sustainabledevelopment.un.org/sdg7.

2 Kuznets (1955) suggests that income inequality initially rises as income per capita rises with increased market forces. However, income inequality begins to decrease after a certain level suggesting a decline in income inequality in the long run (Dogan et al., 2020).

3 However, several studies have suggested an N-shaped relationship (see, e.g. de Bruyn et al., 1998; Poudel et al., 2009; Álvarez-Herranz and Lorente 2016), indicating that environmental degradation will start to rise again beyond a certain income level. Yet, this study does not cover discussions on an N-shaped relationship between CO_2_ emissions and GDP per capita.

4 Surveys of the sizeable EKC literature have been provided by, for instance, Sarkodie and Strezov (2019a, b), Kaika and Zervas (2013a, 2013b), Galeotti (2007), Liu (2005), Frankel and Rose (2005), Dinda (2004), Roberts and Grimes (1997), Panayotou (1993) and Shafik and Bandyopadhyay (1992).

5 The EKC hypothesis also describes emissions as a function of income indicating a unidirectional causality that runs from income to emissions (Acaravci and Ozturk, 2010).

6 Kunnas and Myllyntaus (2007) found support for the EKC hypothesis only for SO_2_ (sulphur dioxide) emissions and, with some reservations, also for nitrogen oxides.

7 That is, 20% increase in energy efficiency, 20% reduction of CO_2_ emissions and 20% renewables by 2020.

8 We followed several previous studies in this connection including Dogan et al. (2020), Halicioglu (2009), Tamazian and Rao (2010), Destek and Sarkodie (2019), Allard et al. (2018), Alvarez-Herranz et al. (2017), Bilgili et al. (2016), Koçak and Ulucak (2019), Li and Lin (2015) and Lin et al. (2016).

9 Many studies have also included a cubic term of Y (i.e. Y^3^) to check whether the EKC curve is N-shaped.

10 For studies that examine N-shaped EKC, expected links are as follows: (i) If β1 > 0 and β2 < 0 and β3 > 0, there will be a cubic polynomial or N-shaped relationship between environmental deterioration and income. (ii) If β1 < 0 and β2 > 0 and β3 < 0, there will be an inverted, or opposite, N-shaped relationship between environmental degradation and economic growth.

11 Chudik and Pesaran (2015) show that when a lagged dependent variable enters the list of regressors (this would then be a dynamic panel data model), only the CCEMG estimator remains consistent under certain conditions.

12 The feedback hypothesis suggests that if sustainable management options are adopted in the production and consumption of natural resources, the rate of natural resource depletion and environmental stress declines.

13 Variables like renewable energy, energy intensity and electricity consumption have also been used as the proxies for the energy use variable in regressions. Results of such exercises are generally similar. These results are available through personal requests.
